# Comparison of Post-Vaccination Response between mRNA and Vector Vaccines against SARS-CoV-2 in Terms of Humoral Response after Six Months of Observation

**DOI:** 10.3390/vaccines11101625

**Published:** 2023-10-23

**Authors:** Sebastian Slomka, Patrycja Zieba, Oskar Rosiak, Anna Piekarska

**Affiliations:** 1 Department of Internal Medicine and Geriatrics, Biegański Regional Specialist Hospital, 91-347 Lodz, Poland; sslomka@wp.pl (S.S.); patrycjazieba7@gmail.com (P.Z.); 2 Department of Otolaryngology, Polish Mother’s Memorial Hospital, Research Institute, 98-338 Lodz, Poland; orosiak@gmail.com; 3 Department of Infectious Diseases and Hepatology, Medical University of Lodz, 90-419 Lodz, Poland

**Keywords:** SARS-CoV-2, mRNA vaccine, vector vaccine, Comirnaty, Vaxzevria, IgG SRBD antibody, COVID-19

## Abstract

Background: The emergence of the SARS-CoV-2 (COVID-19) pandemic has accelerated work on the creation of effective vaccines, both in terms of previously known vector vaccines and new-generation (mRNA) vaccines. The scientific research on vaccination against COVID-19 infection is limited; therefore, understanding how the immune system responds to vaccines is critical. In our study, we conducted a long-term analysis of the presence and persistence of the immune response via chemiluminescence, analyzing the level of IgG antibodies and neutralizing antibodies in subjects vaccinated with two types of mRNA (Comirnaty) and vector (Vaxzevria) vaccines. Materials and methods: Healthcare workers and a group of teachers were recruited for this study according to the 2021 government-launched vaccination calendar. They received two doses of the Comirnaty or Vaxzevria vaccine. SRBD (spike-receptor binding domain) IgG antibody levels were measured monthly for 6 consecutive months with a chemiluminescent assay (CLIA) and neutralizing antibodies for two periods—1 and 5 months from the completion of the vaccination course. Results: 168 people were recruited for this study: 135 people for the mRNA vaccine group and 33 people for the vector vaccine group. Comparing the serum IgG levels between the two types of vaccines, a significant difference in median values can be noted at all time points. In consecutive months, the mRNA-vaccinated group exhibited significantly higher SRBD levels compared to the vector group, with peak concentrations at one month after the complete vaccination cycle (745 AU/mL vs. 15.44 AU/mL; *p* < 0.001). Peak antibody concentration for the vector vaccine was observed one month later, at the third follow-up visit; however, the median IgG concentration was almost 7.7 times higher for the Comirnaty group. Both products were effective in stimulating neutralizing antibody production after vaccination. Higher median values were observed for the mRNA vaccines in both evaluations. At first evaluation, the median value for NA concentration in the Comirnaty group was 6 times higher than in the Vaxzevria group (median value 12.23 [IQR 27.3] vs. 1.7 [IQR 3.3]; *p* < 0.001. Conclusions: People vaccinated with the mRNA vaccine (Comirnaty) showed a stronger immune response to the vaccination than the group of people administered the vector vaccine (Vaxzevria). The Comirnaty group showed higher levels of IgG, including neutralizing antibodies, at all time points during the follow-up period, and this was independent of having had a SARS-CoV-2 infection. A natural decrease in antibody levels was seen within 6 months. A booster vaccination may be required. No serious side effects were observed in either group.

## 1. Introduction

The SARS-CoV-2 pandemic greatly accelerated the process of developing mRNA vaccine technology [1]. It was also the first such opportunity to implement this modern method of vaccination on a global scale.

Currently, there are two mRNA vaccines approved by the FDA (Food and Drug Administration) and the EMA (European Medicines Agency): Pfizer-BioNTech Comirnaty and Moderna COVID-19. The mechanism of action of these vaccines is based on exposure to immune cells of a short, synthetically created fragment of a viral RNA sequence that encodes the Spike protein (S protein) [2]. It is located on the surface of the SARS-CoV-2 virus, and it is responsible for binding the virus particle to the ACE-2 receptor. Due to its function, it is a major target for neutralizing antibodies.

There are several potential advantages of mRNA vaccines, including the ease and speed of their production, which is a direct result of their mechanism of action. This shortens the time needed to acquire immunity by directly delivering the transcript encoding the antigen [3]. mRNA-based vaccines are safer than DNA-based vaccines because mRNA does not interact with the genome of the vaccinated patient and does not have the ability to integrate into it [4,5,6]. In addition, mRNA-based vaccines are directly translated through the host’s translational machinery and lack a bacterial or viral vector, resulting in a low risk of adverse vaccine reactions [7]. The most significant disadvantage of mRNA vaccination is its low thermostability, which requires it be prepared under special conditions: ultra-low temperatures for vaccine storage and transport [8]. This is also an expensive undertaking [9], but it is hypothesized that this situation will improve with the development of more and more stable nanolipid carriers to be used in such vaccines [10,11]. The analyzed effect of mRNA vaccination in our work concerns the Comirnaty vaccine [3]. Vector vaccines, in contrast to mRNA preparations, use viruses to deliver genes encoding vaccine antigens into host cells [12]. Pathogen genes that encode specific antigens that trigger a protective immune response are first inserted into the genome of the vector virus. The vaccine provides a vector that infects host cells. Currently, the most used DNA virus vectors are adenoviruses [13]. In the nucleus, the pathogen’s genes are expressed, resulting in the production of antigens.

We can divide vector vaccines into replicating and non-replicating vaccines: replicating vector vaccines infect cells, resulting in antigen production. Secondarily, a vector virus is also created, which can then infect new cells, which then produce more viral antigens, accelerating immunity acquisition. The only currently registered replicating vaccines are the recombinant virus (rVSV) Zaire Ebola vaccine and the live-attenuated tetravalent dengue vaccine. Non-replicating vector vaccines infect cells, leading to antigen production, but in this case, the vector virus does not replicate in host cells [14]. Several COVID-19 vaccines are based on this technology, including Johnson & Johnson/Janssen, Oxford–AstraZeneca and Gam-COVID-Vac (Sputnik V). Oxford–AstraZeneca’s COVID-19 vaccine is an example of a non-replicating-vector-based vaccine that has been conditionally approved for use in many countries. This vaccine uses a chimpanzee adenoviral vector (ChAdOx1, which was based on ChAdY25) and encodes the SARS-CoV-2 spike protein, protein S [15].

### Aim of Study

This study compared the results of the level of anti-S antibodies and neutralizing antibodies observed within 6 months in a group of people vaccinated with the Pfizer Comirnaty vaccine or the Oxford–AstraZeneca Vaxzevria vaccine.

The following research questions were formulated:

Research question 1. What type of vaccine triggers higher levels of neutralizing antibodies and IgG SRBD antibodies? Research hypothesis 1: mRNA vaccines result in a more pronounced humoral response with higher antibody levels.

Research question 2. What is the dynamic of antibody waning over time, and how is it different for vector and mRNA vaccines? Research hypothesis 2: A waning of antibodies is observed during the observation period and is more pronounced for the mRNA vaccines.

Research question 3. What is the relationship between neutralizing antibody levels and SRBD antibodies, and how is it dependent on the type of vaccine received? Research hypothesis 3: There is a positive correlation between neutralizing antibodies and SRBD antibody concentrations. The correlation is stronger for the mRNA vaccine.

## 2. Materials and Methods

The study protocol was approved by the Bioethics Committee of the Medical University of Lodz (RNN/29/21KE). All subjects gave written informed consent. The entire clinical trial was conducted in accordance with the principles expressed in the Declaration of Helsinki.

People qualified for the group who had received the mRNA vaccines were recruited from the medical and non-medical staff of the University Clinical Hospital No. 1 of the Medical University of Lodz, because healthcare workers were the first in the European Union to be vaccinated with the mRNA vaccine. The people who were qualified to administer the vector vaccine were teachers from secondary schools in Lodz, who, according to the government vaccination campaign, were qualified for vector vaccination and were the second profession to receive the vaccination. Candidates who met all the inclusion criteria and none of the exclusion criteria were included in the study. The study was conducted from January to August 2021. At the time of the study, the recommended vaccine schedule was to administer the second dose of vaccine 1 month after the first dose for Comirnaty and about 2 months for Vaxzevria.

The inclusion criteria were adults aged 18–80 without contraindications to vaccination (fever or symptoms of infection up to 14 days prior) and willingness to report for follow-up visits.

Exclusion criteria were upper respiratory tract infection within the 14 days prior to vaccination, inability to give informed consent, history of diabetes mellitus, bronchial disease or chronic obstructive pulmonary disease.

The study included adult patients of both sexes aged 18–80 who were qualified for vaccination: 133 subjects in the Comirnaty group (mRNA vaccine) and 33 subjects in the Vaxzevria group (vector vaccine).

For those meeting the inclusion criteria and the none of exclusion criteria, blood collection was performed according to the protocol. Blood samples were collected at the first visit (eligibility for vaccination and administration of the first dose) and then every 30 days for 6 months.

In addition, each study participant was given two questionnaires to complete regarding the side effects of the vaccine after the first and second doses. There are 14 symptoms in the questionnaires: low-grade fever, fever, cough, shortness of breath, muscle pain, headache, weakness, rhinitis, stuffy nose, nausea, vomiting, diarrhea, concentration disorders, and taste and smell disorders. The participants rated each symptom on a scale of 0 to 3 based on subjective feedback. The researchers then assigned the participant to one of the following categories: 0 points—asymptomatic; 1–14 points—mild course; 15–25 points—moderate course and >25 points—severe course. The surveys are complementary materials and were designed to investigate the relationship between the severity of the disease and the number of vaccine antibodies.

### 2.1. Description of Laboratory Methodology

Two types of tests were used to analyze the response occurrence to vaccination. The first test was used to quantify S-RBD IgG antibodies to SARS-CoV-2 in vitro in human serum or plasma. In this trial, we used the MAGLUMI^®®^ SARS-CoV-2 S-RBD IgG CLIA (New Industries Biomedical Engineering Co., Ltd. (Snibe), Shenzhen, China), which was granted Emergency Use Authorization by the US Food and Drug Administration and anti-NCP during the initial months of the pandemic, followed by its use as an anti-S-RBD later in 2020.

To assess the number of SARS-CoV-2-neutralizing antibodies, we used the MAGLUMI SARS-CoV-2-Neutralizing Antibody CLIA test (New Industries Biomedical Engineering Co., Ltd. (Snibe), Shenzhen, China). Serum samples (2 × 4.9 mL) were taken from the healthcare workers and teachers after they signed the consent forms. The serum was collected by centrifuging the samples at 4000× *g* relative centrifugal force for 5 min. Serum samples were stored at −20 degrees Celsius until analysis.

### 2.2. Statistical Analysisis 

Data were stored on a computer and analyzed using Statistica 13.3 Software (TIBCO Software Inc., Palo Alto, CA, USA). Nominal variables were compared between the groups in contingency tables using the chi-squared Yates test and Fisher’s exact test for cell counts fewer than 5. Continuous variables were tested for normality of distribution using the Shapiro–Wilk test. An alpha level of 0.05 was established. For non-normally distributed variables, the median and interquartile range (IQR) were analyzed, while for normally distributed variables, the average and standard deviation (SD) were analyzed. A two-way Wilcoxon test was used for comparing paired samples, the Mann–Whitney U test was used for non-paired comparisons, and the Friedman ANOVA was utilized for multiple pairwise group comparisons; these were then followed by a Nemenyi post hoc test. The criterion of statistical significance at *p* < 0.05 was used in the statistical analyses. All tests were double-sided. To account for correlations between variables, a Spearman rank correlation was computed.

### 2.3. Study Group

The study commenced in January 2021 and finished in August 2021; only one virus variant was present in the Polish population at the time. The subjects of this study came from two professional groups who were among the first to receive the vaccination, and the type of vaccination at this time was limited by profession, as designated by the healthcare authorities. Medical staff were vaccinated with the Comirnaty product, and teachers with the Vaxzevria vaccine. No randomization could be performed at the time of the study due to government requirements.

The study population included 168 patients: 33 males (19.65%) and 135 females (80.35%), as visualized in Table 1. The mean age was 46.02 years (SD 12.46); the youngest participant was 23 years old and the oldest was 69 years old, which is the pre-retirement maximum for healthcare workers in Poland. The mean age of the females was 46.39 years (SD 12.2), while for males, it was 44.52 years (13.56). There was no significant difference in age between the genders (Mann–Whitney U test *p* = 0.484).

The total number of COVID-19 convalescents (patients who presented with an initial elevated anti-RBD IgG count over 1.0) was 68, while the remaining 80 patients did not present an IgG count over 1.0 at initial bloodwork. Comparing the Comirnaty and Vaxzevria groups by initial COVID-19 convalescent status, there were 80 convalescents (59.3%) in the Comirnaty group, while in the Vaxzevria group, there were only 8 individuals (22.9%); this was a significant difference between the groups (Yates chi-squared *p* = 0.007).

## 3. Results

### 3.1. Serum Antibody Level Analysis at Time Points

**Figure 1:**
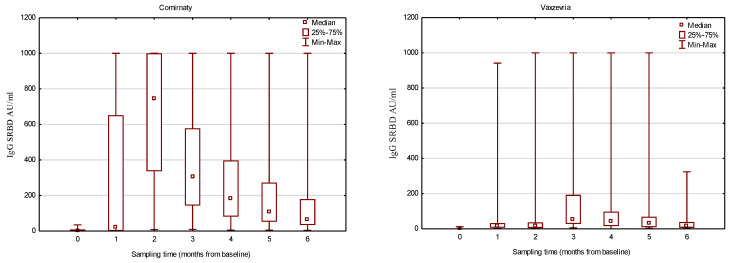
SRBD IgG antibody concentrations in consecutive follow-up visits.

Serum IgG antibody levels were analyzed from baseline to 6 months after the initial visit. A significant increase in SRBD IgG levels was noted for both groups, with different dynamics of response, as visualized in Figure 1.

Comparing the serum IgG levels between the two types of vaccines, a significant difference in median values was noted in all time points except for the first evaluation (on the day of the second dose of the vaccine). In consecutive months, the mRNA-vaccinated group exhibited significantly higher SRBD levels compared to the vector group, with peak concentrations at one month after the complete vaccination cycle (745 AU/mL vs. 15.44 AU/mL; *p* < 0.001). Peak antibody concentration for the vector vaccine was observed one month later, at the third follow-up visit; however, the median IgG concentration was almost 7.7 times higher for the Comirnaty group. Table 2 presents a detailed analysis.

### 3.2. Neutralizing Antibody Synthesis

**Figure 2:**
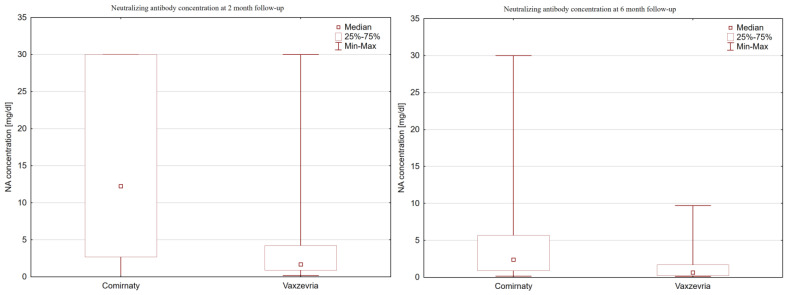
Neutralizing antibody concentrations in consecutive follow-up visits.

Neutralizing antibodies (NAs) were evaluated twice, at a two-month follow-up and at a six-month follow-up, to account for possible waning of the antibody levels. Both products were effective in stimulating neutralized antibody production after vaccination. Higher median values were observed for the mRNA vaccines at both time points, as presented in Table 3. At the first evaluation, the median value for the NA concentration in the Comirnaty group was 6 times higher than that in the Vaxzevria group (median value 12.23 [IQR 27.3] vs. 1.7 [IQR 3.3]; *p* < 0.001), as visualized in Figure 2. On the consecutive follow-up visit, a significant waning of antibody concentration was observed, although the Comirnaty group still exhibited an NA count that was almost 4 times higher. The dynamics of antibody waning were more visible in the mRNA group.

The exact association between IgG SRBD antibodies and neutralizing antibodies is not clear; therefore, we conducted a Spearman correlation analysis and observed significant positive correlations between the SRBD and NA antibodies. The results are presented in Table 4.

### 3.3. Results for SRBD IgG and NA Excluding COVID-19 Convalescents

**Figure 3:**
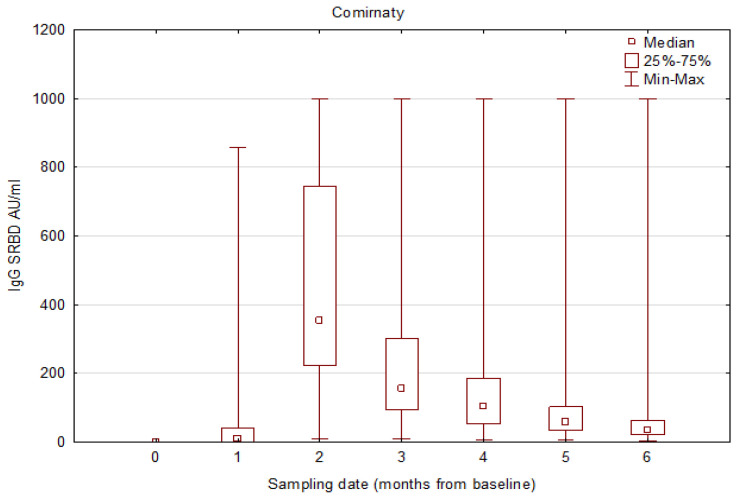
IgG SRBD antibody concentrations in consecutive follow-up visits, excluding COVID-19 convalescents in the Comirnaty-vaccinated group.

**Figure 4:**
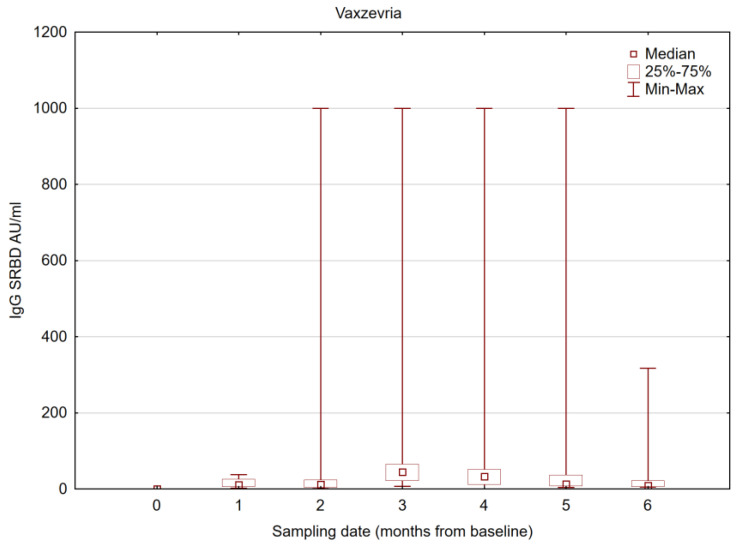
IgG SRBD antibody concentrations in consecutive follow-up visits, excluding COVID-19 convalescents in the Vaxzevria-vaccinated group.

Given that COVID-19 convalescents show a more dynamic mRNA vaccine response and have significantly higher post-vaccination SRBD IgG concentrations than the non-convalescent group [16], we performed an additional analysis excluding all patients who were convalescent from COVID-19. We proved a negligible effect of this factor (Table 5 and Table 6; Figure 3 and Figure 4).

### 3.4. Patient-Reported Post-Vaccine Side Effects

There were significant differences in side effects reported regarding the severity of post-vaccination symptoms after the first dose of the vaccine. More individuals reported symptoms in the mRNA group, with a significantly higher percentage reporting mild symptoms (43% vs. 12.1%) in the self-report questionnaire (*p* = 0.001). After the second dose, the reaction was the same in both groups. Table 7 presents a detailed analysis.

## 4. Discussion

The results of our study show statistically significant differences in antibody levels after vaccination with the mRNA and the vector vaccines. In both cases, the highest antibody values were recorded during follow-up one month after vaccination with the second dose of vaccine. An important observation is that the highest level of SRBD antibodies induced by the Comirnaty vaccine was 13.3 times greater than the level induced by the Vaxzevria vaccine. The mean values of antibody levels induced by mRNA vaccination were almost 7.7 times higher. These differences had decreased by 6 months after the first dose. However, in people vaccinated with the mRNA vaccine, the level of antibodies was still 3.6 times higher than in the group vaccinated with the vector vaccine (66.53 vs. 18.58 AU/mL).

A similar study by Sughayer et al., who compared the level of anti-RBD antibodies induced by different SARS-CoV-2 vaccines based on a SARS-CoV-2 IgG II Quant chemiluminescence test of 2065 blood samples collected from donors, showed that the average level of antibodies induced by Comirnaty was over 3 times higher than the level of anti-RBD antibodies produced in response to the Vaxzevria vaccine [17].

In their study, the neutralizing antibodies were also higher on follow-up in the mRNA-vaccinated group. The level of these antibodies recorded 2 months after vaccination was, on average, 12.23 mcg/dL for Comirnaty vs. 1.7 mcg/dL for Vaxzevria; this is over 7 times higher after mRNA vaccination. At the second follow-up, although the difference in NA levels between the groups was smaller, Comirnaty vaccination still maintained 3.42-fold higher levels.

In line with the assumptions of hypothesis 2, we observed a decrease in antibody levels in both groups of patients vaccinated with different preparations. As already mentioned, the highest level of anti-SRBD antibodies induced by the two vaccines was recorded during follow-up one month after the second dose. However, the greatest decrease in antibody levels during the Comirnaty vaccination was observed just 2 months after the second dose was administered—a more than two-fold decrease (from 745.7 AU/mL to 306.9 AU/mL). The absolute difference between the highest recorded antibody level and the lowest (recorded in the 6th month of observation) was 679.17 AU/mL—a more than 11-fold decrease. Similar dynamics of the decrease in antibody levels for the Comirnaty vaccination were also observed by other authors of similar studies [18,19].

In the case of Vaxzevria vaccination, the greatest difference in antibody levels was observed in the 2nd month after administration of the second dose of the vaccine. However, this was significantly less dynamic than in the mRNA group, with only a 0.72-fold increase in SRBD levels. The absolute difference between the highest and lowest recorded antibody levels was 37.21 AU/mL—a 3-fold decrease. Based on the above information, it can be concluded that despite higher values of Comirnaty-induced antibody levels, the dynamics of antibody level decline is lower for the Vaxzevria vaccine. Our observations are in line with the research of Sughayer et al. [17], where similar changes in antibody levels in patients vaccinated with different preparations were observed.

The observation period should be extended to establish further dynamics of changes in antibody levels in both groups. A similar situation concerns the dynamics of changes in the level of neutralizing antibodies.

The correlation between the level of antibodies and their dynamics, as well as the effectiveness of vaccination, is one of the most important factors validating the relationship between the levels of SRBD and neutralizing antibodies after vaccination with various preparations. In this study, the effectiveness of vaccinations is assessed differently compared to most studies based on the analysis of the risk of infection and the course of the disease after using a given type of vaccine [20,21,22,23,24,25,26,27]. Immunization with mRNA preparations was found to be highly effective by Polac et al. [20] regarding the BNT162B2 vaccine (Comirnaty) and by Baden et al. [21] regarding the mRNA-1273 vaccine (Moderna), where both vaccines exhibited a vaccine efficacy at over 94%.

In vector vaccine studies, only Logunov et al. [22] demonstrated high efficacy of the RAD26/RAD5 vector vaccine (Sputnik), at up to 91.6%, which was not achieved in the overall analysis of the ChAdOx1 vaccine (Vaxzevria) in the study by Voysey et al. [23]. The effectiveness of this vaccine was established at 70.4%. Only in a subset of people in the UK study who received the reduced single dose was the efficacy rate 90% [23]. However, further research is needed to link this effectiveness with a statistically significant difference in the levels of antibodies induced by mRNA and vector vaccinations.

Our previous study [16] established that the group of COVID-19 convalescents exhibited a more dynamic response to the mRNA vaccine and had significantly higher SRBD IgG concentrations post-vaccination than the non-convalescent group. In this study, we also showed that in the Comirnata group, the antibody rate in convalescents was significantly higher. However, when we conducted an additional analysis that excluded all patients who were COVID-19 convalescents, the influence of this factor was negligible.

Additionally, when the number of women and men participating in the study is compared, there is a large disparity. The number of women who participated in the study was much greater than the number of men, which was also true of the SIREN study [24]. It also concerned healthcare workers, and women accounted for 84% of the cohort. This can be partially attributed to the fact that, according to the official National Health Service data in the UK, 77 percent of the workforce in healthcare is female. Female participants comprised 83% of our study.

Initial observations of 282,000 patients vaccinated with Comirnaty and 345,000 vaccinated with Vaxzevira by Menni et al. showed that systemic side effects, i.e., headache, malaise, and diarrhea, were observed more often in patients vaccinated with the first dose of Vaxzevria (33.7% of the group experienced side effects) than those receiving the first dose of the Comirnaty vaccination. Only 13.5% of participants experienced side effects after the first dose, and 22% after the second dose. However, local reactions, such as pain at the injection site and swelling, were recorded more often in patients vaccinated with Comirnaty; after the first dose, 72% of patients experienced local side effects, and after the second dose, 68.5%. By comparison, after Vaxzevira vaccination, 58.7% experienced local side effects. In the presented study, side effects were more pronounced after the first dose in the group vaccinated with the mRNA vaccination than in the group vaccinated with the vector vaccine. This was one of the greatest differences observed compared to other studies [20] and probably resulted from the number of participants.

## 5. Limitations

This study has several limitations. First, the inherent characteristics of a single-center, prospective cohort study may limit the generalizability of the results. Second, the cohorts vaccinated with different vaccines differed in size and individual characteristics. Most of them were healthcare workers, with more women than men in both groups.

Third, the vaccinated healthcare workers underwent more PCR tests for SARS-CoV-2 infection than the vaccinated teachers.

And fourthly, the vaccination schedule was established by the Ministry of Health based on the manufacturer’s data. The assessment at given time points was aimed at assessing which vaccination model in each period is the most effective and which type of vaccination provides the greatest protection against potential infection.

Even though the mRNA group had already received both doses during the two-month FU, and the vector vaccine group was receiving its second dose, we felt that a specific time frame should be established for both groups to assess antibody levels, regardless of the time between doses.

## 6. Conclusions

Based on the results of the presented study, one can hypothesize that to quickly and effectively counteract COVID-19 through global vaccination of the population, mRNA-based vaccines have shown a greater ability to create a protective response in humans against infectious diseases. In our study, we showed the differences in the speed and level of antibodies produced between the new generation of the mRNA vaccine and a standard vector vaccine. An additional aspect that supports this type of vaccine is the speed at which mRNA-based vaccines can be developed and modified.

## Figures and Tables

**Table 1:** General characteristics of the study population.

Group	Comirnaty (n = 135)	Vaxzevria (n = 33)
Age	Median 45 (min. 23; max. 69)	Median 49 (min. 26; max. 69)
Gender	23 males (17.04%)/112 females (82.96%)	10 males (30.3%)/23 females (69.7%)
Race	N = 135 Caucasian (100%)	N = 33 Caucasian (100%)
Comorbidities	Hypertension (22/16.3%), obesity (16/11.8%), pre-diabetes (7/5.2%)	Hypertension (4/12.1%), obesity (3/9.1%), pre-diabetes (1/3%)

**Table 2:** The serum levels of SRBD IgG antibodies in the Comirnaty- and Vaxzevria-vaccinated groups at baseline and follow-up visits.

Visit No.	Comirnaty (n = 135) IgG SRBD AU/mL		Vaxzevria (n = 33) IgG SRBD AU/mL		Mann–Whitney U Test	
	Median	IQR	Median	IQR	*p*-Value	Z Score
1 (1-month FU)	20.98 ^1^	651.400	16.600	25.708	0.830	−0.217
2 (2-month FU)	745.700	663.100	15.440 ^2^	31.670	<0.001	7.634
3 (3- month FU)	306.900	434.600	55.790	165.020	<0.001	5.578
4 (4-month FU)	183.800	315.460	40.685	81.150	<0.001	5.289
5 (5-month FU)	105.400	219.840	32.290	58.960	<0.001	5.133
6 (6-month FU)	66.530	145.140	18.580	32.830	<0.001	5.266

FU—follow-up; IQR—interquartile range: ^1^ after the second dose of Comirnaty; ^2^ after the second dose of Vaxzevria.

**Table 3:** Serum-neutralizing antibody levels in the Comirnaty- and Vaxzevria-vaccinated groups at two- and six-month follow-up visits.

Visit No.	Comirnaty (n = 135) NA Concentration mcg/mL		Vaxzevria (n = 33) NA Concentration mcg/mL		Mann–Whitney U Test	
	Median	IQR	Median	IQR	*p*-Value	Z Score
1 (2-month FU)	12.230	27.300	1.701	3.309	<0.001	5.178
2 (6-month FU)	2.396	4.769	0.660	1.470	<0.001	4.547

NA—neutralizing antibody; FU—follow-up; IGR—interquartile range.

**Table 4:** Spearman rank correlation results for neutralizing and SRBD antibody concentrations at consecutive follow-up visits.

SRBD IgG Concentration	NA Concentration at One Month after Second Dose		NA Concentration at Six-Month FU	
	Comirnaty (r = )	Vaxzevria (r = )	Comirnaty (r = )	Vaxzevria (r = )
one-month FU	0.21 *	0.52 *	0.20 *	0.45 *
two-month FU	0.74 *	0.61 *	0.72 *	0.55 *
three-month FU	0.76 *	0.97 *	0.80 *	0.84 *
four-month FU	0.72 *	0.96 *	0.88 *	0.93 *
five-month FU	0.71 *	0.92 *	0.89 *	0.96 *
six-month FU	0.68 *	0.90 *	0.89 *	0.95 *

* Correlation significant at *p* < 0.05, NA—neutralizing antibody, FU—follow-up.

**Table 5:** The serum levels of SRBD IgG antibodies in the Comirnaty- and Vaxzevria-vaccinated groups at baseline and follow-up visits, excluding COVID-19 convalescents.

Visit No.	Comirnaty (n = 55) IgG SRBD AU/mL		Vaxzevria (n = 25) IgG SRBD AU/mL		Mann–Whitney U Test	
	Median	IQR	Median	IQR	*p*-Value	Z Score
1 (1-month FU)	9.2 ^1^	41.5	11.2	20.5	0.278	−1.093
2 (2-month FU)	352.5	52.7	11.5 ^2^	19.6	<0.001	6.453
3 (3-month FU)	154.6	20.6	44.7	43.6	<0.001	4.469
4 (4-month FU)	102.9	131.9	33.2	39.5	<0.001	4.256
5 (5-month FU)	59.6	68.7	12.8	29.4	<0.001	4.285
6 (6-month FU)	35.6	43.2	8.9	16.2	<0.001	4.318

FU—follow-up; IQR—interquartile range: ^1^ after the second dose of Comirnaty; ^2^ after the second dose of Vaxzevria.

**Table 6:** Serum-neutralizing antibody levels in the Comirnaty- and Vaxzevria-vaccinated groups at one- and five-month follow-up visits, excluding COVID-19 convalescents.

Visit No.	Comirnaty (n = 55) NA Concentration mcg/mL		Vaxzevria (n = 25) NA Concentration mcg/mL		Mann–Whitney U Test	
	Median	IQR	Median	IQR	*p*-Value	Z Score
1 (2-month FU)	2.54	3.92	1.48	1.55	0.003	2.918
2 (6-month FU)	0.90	1.35	0.45	0.78	0.003	2.948

NA—neutralizing antibody; FU—follow-up.

**Table 7:** Reported post-vaccination symptoms in the Comirnaty- and Vaxzevria-vaccinated groups.

Symptom Severity	Symptom Severity after 1st Dose			Symptom Severity after 2nd Dose		
	None	Mild	Moderate	None	Mild	Moderate
**Comirnaty (n = 135)**	73 (54%)	58 (43%)	4 (3%)	53 (39.3%)	79 (58.5%)	3 (2.2%)
**Vaxzevria (n = 33)**	28 (8.9%)	4 (12.1%)	1 (3%)	12 (36.4%)	20 (60.6%)	1 (3%)
Fischer’s exact test with Freeman–Halton extension for 3 × 2 contingency tables	*p* = 0.001			*p* = 0.869		

## Data Availability

The data presented in this study are available on request from the corresponding author.
